# Time Trend of the Male Proportion at Birth in Brazil, 1979–2004

**DOI:** 10.3390/ijerph6082193

**Published:** 2009-08-12

**Authors:** Gerusa Gibson, Luciana Scarlazzari Costa, Sergio Koifman

**Affiliations:** Post-graduate Program in Environment and Public Health, National School of Public Health, Oswaldo Cruz Foundation, Ministry of Health, Rio de Janeiro, Rua Leopoldo Bulhões, 1480, Manguinhos, Rio de Janeiro, RJ, CEP 21041-210, Brazil; E-Mails: gibsonge@ensp.fiocruz.br (G.G.); scarla@usp.br (L.S.C.)

**Keywords:** birth sex ratio, male proportion at birth, endocrine disrupters, time trend

## Abstract

Several studies suggest that exposure to environmental endocrine disrupters can reduce the number of male births, and therefore, the male proportion at birth (also denominated birth ratio or sex ratio at birth) can be used as a sentinel health indicator. This work aimed to analyze the trend of male births in Brazil, according to their distribution by states and capitals. An ecological descriptive time series was carried out using polynomial regression, showing a declining trend for male proportion at birth in Brazil (1979–1994), followed by an upward trend until 2004. A decline on the proportion of male births was observed in Brazil between 1979 and 1993, followed by a subsequent rise of this ratio between 1995 and 2004, wherein the mean proportion of male births in Brazil rose from 51.05 to 51.18, representing a relative increase of 0.25%. The states of São Paulo (Southwest region) and Acre (Western Amazon), as well as some capitals–Cuiabá (Western Region), Palmas (Amazon) and Rio Branco (Amazon)–showed increasing trends, which suggests the influence of socio-demographic changes. In contrast, a declining trend in the State of Ceará State (Northeast region), with a 0.35% yearly decrease was observed. As a whole, these results suggest the influence of different environmental factors (demographic changes, public health services distribution, and population exposure to endocrine disruptor substances) influencing the time trend of birth ratio in the Brazilian population during the last decades.

## 1. Introduction

The male proportion at birth, also referred as birth ratio or sex birth ratio (male births/all births × 100), is a standard measurement to assess the distribution of birth frequencies according to gender. The natural sex distribution at birth is estimated to be close to 106 males for every 100 females, which yields a male birth proportion of 51.5%.

The excess of male births in human populations is a natural process, arising from the evolution of adaptive mechanisms of nature, as a way to compensate male lower life expectancy comparatively to that observed among women [1]. However, male and female births frequencies may be influenced by different variables, such as genetic factors, environmental exposure to chemical pollutants, and also demographic and social context related factors [2,3].

The factors influencing the male proportion at birth are classified as: primary, including those determining the fetal gender at the time of conception; and secondary, which in turn, influence the survival of the embryo in the maternal uterus, and thus, are related to the neonatal vulnerability [4]. According to this classification, parental hormonal concentration right before conception is considered to be a paramount factor influencing the sex of the embryo [5,6]. As a consequence, the relative frequency of male births is directly related to the integrity of the parental endocrine system in a given population [5].

High gonadotrophin and progesterone serum levels are supposed to increase the probability of a female child birth, which implies a higher ratio of liveborn girls, and a lower proportion of male births. On the other hand, high parents’ estrogen and testosterone serum concentrations at the time of conception seem to increase the probability of a male child birth [5–7].

Other studies also suggest the influence of factors such as parental age, birth order, and ethnic differences, with the sex ratio at birth tending to be higher among Asian populations, and lower among Afro-descent populations [2]. However, the long term effects following environmental contamination subsequent to human interventions have indeed given rise to considerable scientific and public concern [8].

The increasing exposure to xenobiotics released in the environment by industrial and agricultural activity (chemical pollutants that are able to disrupt the hormonal system in humans and wildlife), has been identified as one of the main reasons responsible for the lower frequency of males births in some countries [9–11]. Therefore, some hormonal related pathologies such as male birth defects (hypospadia and cryptorchidism), infertility, sperm quality disturbances, hormonal-related cancers, among others, have been reported as resulting from the influence of endocrine disrupters in the endocrine system homeostasis [9,10].

Several studies have demonstrated the decline in the proportion of male births following both accidental and occupational exposures to endocrine disrupters. The assessment of a cohort exposed to 2,3,7,8-tetrachlorodibenzo-*p*-dioxin (TCDD) after the accident at a chemical plant in Seveso (Italy) showed that high serum concentrations of this compound were significantly associated to a relative increase in the number of female births [12,13]. Furthermore, some population-based studies have reported trends of declining male births in different countries [11,14–17].

The increasing development of industrial and agricultural activities over the years associated with limited environmental sustainability policies, have promoted the introduction of persistent chemical waste in the environment. As a result, bioaccumulation of these substances over time in the various environmental matrices can be foreseen [18]. This new environmental scenario, as well as the growing incidence of infertility and reproductive disturbances in recent years, have triggered an increased interest on the potential effects resulting from chronic exposure to endocrine disrupters [8].

A recent report on the time trend of the male proportion at birth in municipalities of the state of Paraná, Brazil, in which the spread increase in the use of pesticides due to large scale agriculture has played an important role in the last decades, revealed a sharp decline of male births observed since the nineties [10]. Although there is a huge demand for studies evaluating the temporal trend of male proportion in Brazil, there are only a few of them, which reflects the need for studies addressing such issue. In this sense, the objective of the present investigation is to characterize the time trend of the male proportion at birth in the different regions of the country.

## 2. Methodology

### 2.1. Study Design and Data Source

**Figure 1.**
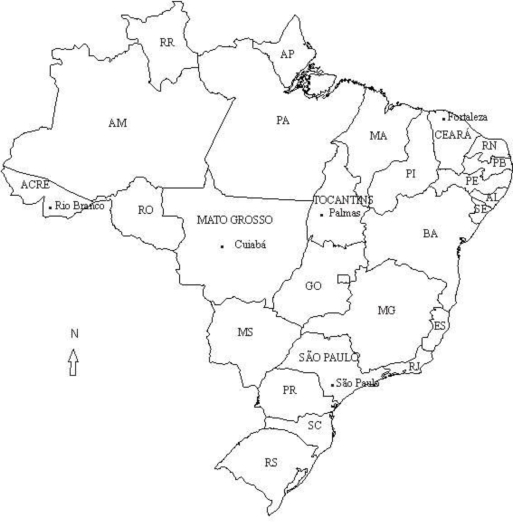
Map of Brazil (selected states and cities mentioned in the text).

An ecological epidemiologic study was carried out with birth data provided by the Brazilian National Health System (SUS) databank (DATASUS), with universal coverage in the country. The yearly proportion of male births was ascertained along 1979–2004 for the whole country, and during 1994–2004 in selected states and capitals (Figure 1). According to all Brazilian births, the male proportion at birth in 1994 was an outlier for unknown reasons. Hence, when analyzing the whole country data, 1994 data was not included, and 1979–1993 and 1995–2004 time intervals were used instead.

### 2.2. Time Trend Analysis

The trend analysis was performed using polynomial regression models, since they offer ease of formulation and interpretation, as well as high statistical power. The polynomial regression models were designed to find the equation which best described the relationships between the dependent (sex ratio at birth) and the independent (calendar years) variables. Hence, the following polynomial regression models were tested, wherein X and Y represent, respectively, the independent and dependent variables, and β0, β1 and β2 are regression coefficients:

- Linear model (1st order): Y = β0 + β1 X
- Second order model: Y = β_0_ + β_1_ X + β_2_ X^2^
- Third order model: Y = β_0_ + β_1_ X + β_2_ X^2^ + β_3_ X^3^

where Y = proportion of male births, X = year, and β0 = mean proportion of male births in the period.

The choice of the most appropriate model was based on three parameters: the analysis of the scatter plot, the analysis of the coefficient of determination (the closer to 1, the most adjusted model) as well as the analysis of the residuals. In situations where two models were similar in terms of statistic power, the model of lower order was chosen. Aiming to avoid the serial correlation between the terms of the equation, the midpoint of the time series, instead of X values, was used.

In this case, for the period from 1994 to 2004, the term (X-1999) represents the central variable. As an example, the linear model has the following expression:

Y = β_0_ + β_1_. (X-1999), where:
Y = Proportion of male births
X = Year
β_0_ = Mean proportion of male births in the period
β_1_ = Annual mean increment

A trend was considered statistically significant when the ascertained regression model showed p-values < 0.05. For models whose p-values were higher than 0.10, the trends were considered stable along the period. The program used to compile the polynomial regression models and the scatter plots was SPSS for Windows (9.0).

## 3. Results

**Figure 2.**
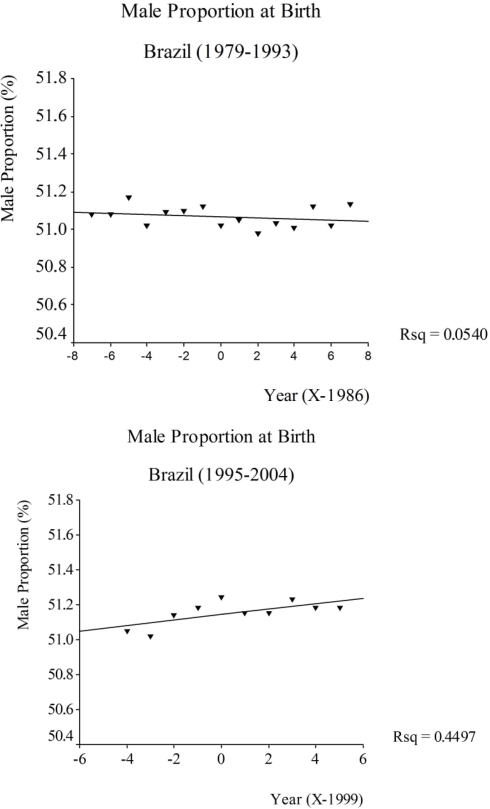
(A) Time trend of male proportion at birth, Brazil, 1979–1993; (B) Time trend of male proportion at birth, Brazil, 1995–2004.

A decline on the proportion of male births was observed in Brazil between 1979 and 1993, followed by a subsequent rise of this ratio between 1995 and 2004. The male proportion at birth in Brazil decreased from 51.08 in 1979 to 51.02 in 1993, representing a relative decline of 0.11% (Figure 2A). In 1995, it was 51.05 and it rose to 51.18 in 2004, which represented a relative increase of 0.25% (Figure 2B). The temporal trends of the proportion of male births in Brazil (1995–2004) and selected states and capitals (1994–2004) are presented at Table 1.

In relation to the States distribution, only the states of Sao Paulo and Acre showed growing significant trends, while Ceará State at the Northeast Region was the only one to present a statistically significant declining trend during the same studied period. The other Brazilian states showed stable trends with no statistically significant changes.

The mean proportion of male births in the State of Sao Paulo (Southeast Region) was 51.17, with an increment of 0.01 per year. In 1994, the State of São Paulo showed a proportion of male live births of 51.15, which increased to 51.18 in 2004, an increment of 0.05%.

**Figure 3.**
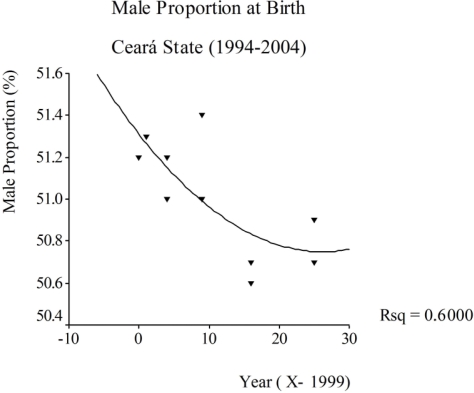
Time trend of male proportion at birth, Brazil, Ceara State, 1994–2004.

The state of Acre (Western Amazon) showed a mean proportion of male births of 51.28 during the period, with an increment of 0.16%. In this state, the mean proportion of male births rose from 49.80 in 1994 to 51.46 in 2004, an increase of 3.3%. In contrast, Ceará State had a mean proportion of male births of 51.30, a negative growth of 0.04 per year. In 1994, the proportion for this state was 50.88 decreasing to 50.70 in 2004, a declining of 0.35% (Table 1; Figure 3).

**Figure 4.**
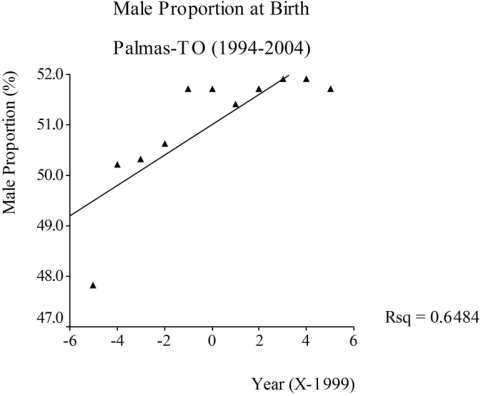
Time trend of male proportion at birth, Brazil, Palmas-TO, 1994–2004.

For Brazilian capitals, only three of them showed statistically significant increasing trends: Cuiabá, Palmas and Rio Branco. The city of Cuiabá (Central Western Region) had a mean proportion of male births of 51.43 and an annual increment of 0.16 for the period. In 1994 this proportion was 49.58 and increased to 51.95 in the year 2004, an increment of 4.8%. In Palmas (Southern Amazon), the mean proportion of male births was 50.99, and an annual increment of 0.3 for the period of study was observed. In 1994, the proportion was 47.78 and increased to 51.70 in 2004, an increment of 8.2% for entire period (Figure 4).

Regarding the city of Rio Branco, capital of the State of Acre, a mean proportion of male births of 51.08 and a yearly increase of 0.22 along the series were verified. In 1994, this proportion was 49.24 and rose to 52.18 in 2004, an increase of 5.6% in the period.

The other Brazilian capitals showed no significant changes in their trends, suggesting that they remained stable for the period.

## 4. Discussion

Ecological studies are often employed in analysis of time series, since they may generate hypotheses and contribute to enhance the understanding on different conditions affecting population health status. Several studies have shown that the male proportion at birth has declined in some countries of Europe and even in the United States and Canada, which has been the subject of intense academic discussion [11,14–16]. In the present study, however, this trend was only observed at the State of Ceara from 1994–2004, but not for the Brazilian population as a whole.

According to Feitosa and Krieger, who used samples of hospital records of live births and stillbirths from 11 countries of Latin America, a statistically significant declining trend of male births in Brazil was observed from 1967 to 1987, as well as in other countries, except for Peru and Uruguay. The authors suggested that this decline could be related to the distribution of stillbirths in these countries, whose incidence was increasing up to 1984 [4].

This paper presents the Brazilian birth series between 1979 and 2004, the longest with available data for all municipalities in the whole country. In order to evaluate the impact of an outlier (1994) on the whole series, the distribution of male proportions at birth was also ascertained excluding such extreme value, which does not change the trend, but remains less accentuated. No apparent reason for the occurrence of such outlier could be identified, since the procedures for recording live births in the country, which are consolidated centrally at the Ministry of Health, had not changed along the series.

The observed distribution of male proportions at birth was in agreement with results in the literature [4], since the same declining trend was observed (Figure 2A). These findings suggest that, until 1994, Brazil presented a pattern similar to that observed in Canada and the United States between 1970 and 1990 [14,17]. The same declining trend was also observed in Denmark from the 50s, and in Sweden, Finland, Germany and Norway from the 70s, a period of intensive industrial development in these countries [15]. Since 1994, however, the trend started to increase until the end of the series in 2004, also less marked when the year of 1994 is removed from analysis (Figure 2B).

It should be highlighted that the decline in the frequency of male births in Europe, as well as in the United States and Canada in the second half of the Twentieth Century, was not restricted to these countries, since the phenomenon was also observed in Brazil and several countries of Latin America [4]. It must also be emphasized that this phenomenon occurred in a historical context marked by a marked industrial development, especially in the chemical industry [15].

For Brazilian states and capitals, most of them did not show any significant variations, suggesting that the sex ratio at birth has remained stable over the period 1994 to 2004. However, the increase observed in the State of Acre and also in the capitals Palmas, Cuiabá and Rio Branco (all of them located in areas characterized by low industrial activities), rose from values considered below the usual (51.0) in 1994, to values over 51.0 in 2004. All these capitals have received a large migration wave during the last decades following the agriculture frontier expansion, an important demographic process supported by the Brazilian government and mainly including migrants from the Southern States (Rio Grande do Sul-RS, Santa Catarina-SC and Parana-PR, Figure 1) settled in the Amazon.

Regarding São Paulo State, the increasing trend of male proportion at births, although statistically significant, does not seem to have great impact on the final mean of the period, since the variation was too small. It is important to mention that this State has a large community of Asian immigrants, an ethnic group whose sex ratio tends to be higher in comparison with other groups [2].

Although the declining trend observed at the State of Parana has shown no statistical significance, it has a pattern of intensive agricultural activity, and therefore, showing a massive use of several pesticides, chemicals substances with acknowledged characteristics of environmental endocrine disrupters.

In relation to Ceará State, which showed a declining trend, it was observed that the proportion of male births was 50.7 in 2004, a lower borderline compared to normal values described in the literature, which deserves attention in the following years [2]. Moreover, this state has been presenting an important growth in the last decades on large scale agriculture in rural areas for commodities (mainly fruits) production and exportation, with intensive use of pesticides, which may have influenced the observed male proportion at birth.

Among the five capitals that showed statistically significant growing trends, Palmas presented the greatest increase in the period (8.2%), followed by Rio Branco and Cuiabá, which had 5.6% and 4.8% ratios, respectively. This suggests that both Acre State and its capital Rio Branco showed similar trends, a fact that may be occurring in other municipalities of this Amazonian state.

It deserves to be mentioned that the significant growing trend observed in some states and capitals may just reflect prenatal care services improvement, which could have contributed to reducing fetal deaths, especially male stillbirths [4].

Two other factors must be considered in the interpretation of the obtained results: the reduction of maternal parity observed in the country in recent years, and also the maternal age rise at the first full term pregnancy. According to previous scientific studies, these factors are negatively associated with the sex ratio at birth [2,16].

According to national health data, 33,219,289 births occurred in Brazil from 1994 to 2004, with 16,980,877 registered as male, 16,145,519 as female, and 92,893 with unknown sex at the time of registration. These numbers represent an increase of 17.7% of live births in Brazil, an increase of 18.9% of male births and 18.4% of female births.

Although the use of census data presents limitations, they are useful to trace population features, such as the male proportion at birth. Accordingly, the risk of systematic errors in the reporting of census data should be considered when interpreting the results.

There were a large number of births with unknown sex, especially at the beginning of the series, although this number has decreased 85.9% during the entire series.

Although some studies have reported declining trend of sex ratio at birth in some countries, others have reported stable or even increasing trends [14,17]. For some authors, the use of sex ratio as a sentinel indicator of the environmental health must be questioned, since its sensitivity to detect environmental exposures is not so high [19].

Nevertheless, several studies have reported lower frequency of male births as a result of occupational exposures to environmental endocrine disrupters, especially in case-control studies [20]. On the other hand, when analyzing chronic exposures, the methodological limitations may lead to difficulties in establishing cause-effect relationships, especially in ecological studies [10].

In the case of Brazil, the growing trend of male births in some states and capitals suggests that these differences can be explained by local socio-demographic factors. In the last decades, a reduction of maternal parity has been reported, while the maternal age has increased. Additionally, Brazil has experienced a reduction in the incidence of stillbirths due to improvements in prenatal care [2,22].

These results do not strongly support the hypothesis of exposure to environmental endocrine disrupters, except for Ceará and Paraná State, whose trends were declining. Regarding Palmas city, it is important to emphasize that a great increase in the number of births has been reported since the recent creation of the state of Tocantins in 1988, which received a massive migrant population. From that year, the total number of births in the city has an increase of 133.8%, which confirms the strong growth of the capital.

The sex ratio tends to present larger variation among ethnically different populations [2]. On the other hand, intra-population variation occurs where other variables such as age of relatives, multiple pregnancy, birth order and predisposition of some individuals or couples to have predominantly children of one particular sex (Lexis association), seems to have greater relevance [2,3]. Based on these concepts, it is plausible that the increasing trend observed in Brazil and also in some states and capitals, has resulted from demographic changes which have been occurring in recent years. Moreover, the declining trend observed for Ceará State shows the need for future studies in order to better investigate its causes, since the phenomenon has been linked to exposure to environmental endocrine disrupters [12].

This paper presents methodological limitations, such as not controlling for potential confounding variables. As an ecological study, individual information of individual exposures, such as smoking, reproductive antecedents, medicines intake, among others which may introduce confounding in the observed results, could not be evaluated. Hence, the use of a time series approach enabling autocorrelation control as suggested in the literature [23], could not be performed in this study.

Other restrain was the relative shortness (25 years) of the analyzed time series. Differently than verified in other countries with largest series, the Brazilian analyzed series limits generalization of the observed results. However, as a pioneer initiative in the country trying to describe the recent distribution of male proportion at birth, it allowed to generate hypothesis on the possible environmental conditions potentially associated to the studied phenomenon, which can be further re-evaluated when larger series become available.

Taking into account such restrains, this work represents an initial step for exploring the distribution of sex ratio at birth in Brazil, considering the great scarcity of this type of study in the country. As a whole, the time trend on the proportion of male births seems to present two trends in the last decades: a falling one between 1979–1993, and a rising trend afterwards. The recent demographic and economic features observed in the different regions of Brazil seems to offer some answers to such distinct patterns, including the expansion of public health services, the intensive migration process, and the wide population exposure to chemicals with endocrine disrupter properties, such as pesticides. The study of the distribution of variables associated to sex ratio determination already described in the literature are goals to be achieved in future studies in developing countries such as Brazil, experiencing a rapid demographic and environmental change subsequently to intensive industrialization, urbanization and adoption of large scale agriculture.

## Figures and Tables

**Table 1.** Male proportion at birth, Brazil, selected states and capitals, 1979–2004.

**Region**	**Best fit model**	**Determination Coefficient (R^2^)**	**p-value**	**Trend**

Brazil 1	Y = 51.06 – 0.002X	0.0540	>0.05	Decreasing
Brazil^2^	Y = 51.15 + 0.01X	0.4497	<0.05	Increasing
Sao Paulo State 3	Y = 51.17 + 0.01X	0.4260	<0.05	Increasing
Acre State 3	Y = 51.28 + 0.16X	0.5285	<0.05	Increasing
Ceara State 3	Y = 51.30+0.0008X – 0.04X^2^	0.5999	<0.05	Decreasing
Cuiaba, MT 3	Y = 51.43 + 0.16X	0.4627	<0.05	Increasing
Palmas, TO 3	Y = 50.99 + 0.30X	0.6483	<0.05	Increasing
Rio Branco, AC 3	Y = 51.08 + 0.22X	0.7017	<0.05	Increasing

^1^ Time trend of male proportion at birth, 1979–1993

^2^ Time trend of male proportion at birth, 1995–2004

^3^ Time trend of male proportion at birth, 1994–2004
